# Designing a flipped AI-chatbot learning module to support students’ environmental literacy development: A Fuzzy Delphi Method

**DOI:** 10.1371/journal.pone.0345027

**Published:** 2026-03-18

**Authors:** Xiaoyu Wang, Xiang Li

**Affiliations:** 1 School of Education, Sanda University, Shanghai, China; 2 Department of Curriculum and Instructional Technology, Faculty of Education, University of Malaya (UM), Kuala Lumpur, Malaysia; 3 Department of Environmental Engineering, Hebei University of Environmental Engineering, Qinhuangdao, China; National University of Malaysia Faculty of Education: Universiti Kebangsaan Malaysia Fakulti Pendidikan, MALAYSIA

## Abstract

China’s rapid economic growth has exacerbated environmental degradation, posing severe risks to public health and sustainable development. However, current environmental education in higher education remains predominantly teacher-centered, resulting in low engagement and inadequate development of Environmental Literacy (EL). Correspondingly, this study addresses these challenges by designing a Flipped AI-Chatbot Learning (FACL) module that is designed to support the development of students’ EL through an innovative integration of generative Artificial Intelligence (AI) and Flipped Learning (FL). Using the Fuzzy Delphi Method (FDM), this research gathered the consensus of 12 experts to develop a comprehensive instructional framework grounded in educational theories. In particular, the FACL module combines pre-class AI chatbot interactions with in-class active learning strategies to promote personalized, student-centered learning. Accordingly, the results identify key instructional objectives, strategies, and evaluation mechanisms, specifying module elements intended to address traditional teaching challenges. This includes low motivation, limited interaction, and inadequate pre-class preparation. Concurrently, this study contributes to environmental science, educational technology, and information literacy by providing a scalable and interdisciplinary framework that aligns theoretical innovation with practical application. Overall, the FACL module advances environmental education while potentially cultivating critical thinking and pro-environmental behaviors, with the intention of preparing students to address complex sustainability challenges in real-world contexts.

## Introduction

China’s rapid economic expansion has come at a high environmental cost, resulting in widespread degradation that poses a threat to public health and sustainability. Notably, pollution alone accounts for an estimated 4.2 million deaths annually, underscoring its profound impact on human well-being and the pressing need for sustainable social and economic development [1]. However, addressing these challenges requires a transformative approach to environmental education that emphasizes the development of Environmental Literacy (EL) among future generations.

EL, a cornerstone of environmental education, equips individuals with the knowledge, skills, and attitudes necessary to understand and address environmental challenges. Furthermore, it serves as a foundation for fostering environmental awareness and driving societal transitions toward sustainability and healthier lifestyles [2]. Despite its critical significance, current environmental education in higher education institutions remains inadequate. Predominantly teacher-centered, it fails to engage students actively, resulting in minimal interaction and limited learning outcomes [3]. This inadequacy is reflected in the alarmingly low EL rate of just 17.66% among college students in China [4]. Additionally, students’ environmental knowledge, attitudes, and behaviors are inconsistent and limited. Although awareness levels are relatively high, they often fail to translate into meaningful actions [5]. Moreover, traditional lecture-based courses often emphasize rote memorization. This leads to negative perceptions of these courses as irrelevant to everyday life and contributing to low student motivation and engagement [6,7]. In line with this, teacher-centered methods hinder the development of critical thinking and problem-solving skills, which are essential for addressing complex environmental challenges. Nevertheless, their emphasis on fragmented knowledge further limits students’ ability to apply classroom learning to practical, real-world contexts [8,9]. Additionally, the lack of personalized learning processes prevents students from engaging in self-regulated learning, as one-size-fits-all methods fail to accommodate diverse learning needs [10]. Consequently, this limitation reduces opportunities for interactive and meaningful learning experiences, further diminishing the effectiveness of traditional approaches. Given these perspectives, a new learning module is needed to address these challenges and support motivation and engagement. It should also help cultivate critical thinking and problem-solving, while bridging classroom knowledge with real-world applications, thereby supporting EL development. In particular, this module employs a more student-centered, interactive, and diverse approach to teaching techniques, catering to the needs and expectations of both students and teachers.

A promising foundation for this approach is the flipped classroom model, which reverses the traditional classroom dynamic by delivering instructional material outside of class and dedicating class time to active, collaborative learning [11]. This strategy enables students to be better prepared to engage in conversations, problem-solving activities, and higher-order thinking tasks during class time when it is used [12]. Nevertheless, Flipped Learning (FL) commonly faces challenges, such as inadequate supervision at home and insufficient support during pre-class preparation, which prevent students from actively participating in the classroom [13].

Personalized, interactive, and engaging learning environments provided by Artificial Intelligence (AI) chatbots, such as DeepSeek or ChatGPT, offer promising solutions to these challenges [14]. They also offer prompt feedback, accommodate diverse learning styles, and simplify complex environmental concepts. Thus, by simulating human interactions, AI chatbots make learning more interesting and personalized [15]. Furthermore, these capabilities may help address shortcomings of FL by promoting learning opportunities, increasing interactivity, and supporting students during pre-class preparation. According to Diwanji et al. (2018), utilizing AI chatbots in the flipped classroom effectively addresses these challenges. It provides students with 24/7 personalized support for pre-class tasks, helping them better prepare for in-class activities [16]. Concurrently, AI chatbots enable individualized, self-paced learning outside the classroom while enhancing interactive, guided learning during class sessions [17]. As a result, this type of integration facilitates students’ learning journeys and improves class engagement and interaction.

Although incorporating AI chatbots into FL shows promising potential, the concept is still relatively new. Correspondingly, research on the use of AI chatbots in education and in FL has been conducted separately. However, there has been minimal inquiry into the application of both approaches together [18]. Baskara (2023) highlighted that the use of chatbots in FL is still in its early stages and requires further study [17].

Therefore, these discrepancies underscore the need for an innovative framework to address theoretical and practical gaps in leveraging AI chatbots in FL to support students’ EL development. Following this, expert contributions are vital to developing such a framework and ensuring its rigor, applicability, and effectiveness in meeting contemporary educational needs. Thus, by validating the theoretical foundation, experts align the framework with modern pedagogical principles, ensuring its relevance and applicability. Furthermore, their interdisciplinary expertise ensures that the content is both scientifically accurate and contextually appropriate, addressing the complexities of environmental education. In addition, experts contribute instructional strategies that maximize student engagement and tailor the module to diverse educational contexts, enhancing learning outcomes. They also design robust evaluation mechanisms to assess knowledge acquisition, critical skill development, and behavioral changes, aligning these assessments with the module’s objectives. Beyond design and implementation, experts evaluated the relevance, clarity, and feasibility of the module elements, supporting alignment with its intended goals of fostering EL and addressing real-world environmental challenges. Essentially, this collaboration underscores the crucial role of expert involvement in developing comprehensive and impactful educational frameworks. This is supported by prior research emphasizing the value of expert consensus in course design [19,20].

Consequently, this study develops a Flipped AI-Chatbot Learning (FACL) module based on expert consensus, addressing the shift from teacher-centered to student-centered learning and providing a design framework intended to support EL development. The following questions guide the research: 1. What are the objectives of the FACL module to support students’ EL development based on experts’ opinions?

1. What content does the FACL module include to support students’ EL development based on experts’ opinions?
2. What instructional strategies are employed in the FACL module to support students’ EL development based on experts’ opinions?
3. What instructional resources and platforms are utilized in the FACL module to support students’ EL development based on experts’ opinions?
4. What evaluation strategies are implemented in the FACL module to support students’ EL development based on experts’ opinions?

## Theoretical background

A theoretical framework organizes key concepts and assumptions derived from relevant theories to support a study within a specific context [21]. The FACL module is grounded in four complementary theoretical frameworks. The ‘First Principle of Instruction (FPI)’ is an Instructional Design (ID) theory used to develop the instructional environment. On the other hand, ‘Constructivism Learning Theory,’ ‘Social Constructivism,’ and ‘Situated Cognition Theory’ are Learning Theories (LTs) used to develop learning strategies.

### First principle of instruction (FPI)

This study adopted Merrill’s (2002) FPI as the Instructional Design (ID) framework. FPI emphasizes problem-centered learning and proposes five core principles: activating prior knowledge, demonstrating new content, applying new content in authentic contexts, and integrating new knowledge into learners’ experiences [22,23]. Collectively, these principles highlight the significance of engaging learners in meaningful tasks that connect instruction to real-world problem-solving.

Specifically, Merrill (2002) argued that these principles are applicable across various instructional delivery systems and are particularly effective for addressing real-world issues. In the context of environmental education, FPI supports integrating academic concepts with real-world environmental problems, enabling students to apply their knowledge in authentic situations. Accordingly, FPI was selected as the ID foundation for the FACL module to support students’ EL development.

### Constructivist learning theory

Constructivist Learning Theory suggests that learners actively construct understanding by integrating new information with prior experiences, thereby fostering deep, meaningful learning [24]. Central tenets of constructivism include active learning, knowledge construction, contextual learning, social interaction, mental engagement (both cognitive and physical), and recognition of individual differences among learners [24–26].

These principles emphasize learners’ active involvement in meaning-making and the importance of situating learning within relevant contexts. These characteristics align well with the personalized learning affordances of AI chatbots and the active-learning emphasis of the flipped classroom. Notably, Constructivist Learning Theory provides a coherent theoretical basis for designing EL-oriented learning activities within the FACL module.

### Social constructivism

Social Constructivism, employed in this research to support learning processes, underscores that human growth is socially situated and knowledge is formed through interaction with others [27]. It considers how social interactions and cultural environments affect learning [25]. Key principles include social activity, where interaction, debate, and cooperation with informed individuals enhance learning, the role of language and culture in intellectual growth, and knowledge co-construction. Another key principle is the Zone of Proximal Development (ZPD), or scaffolding, in which support from teachers and peers helps learners progress until they can perform tasks independently [25,28–30].

Additionally, Vygotsky (1968) emphasized the significance of social connections and teamwork in learning, aligning with the FACL module. The module adopts Social Constructivism by facilitating interactions among students, AI, and teachers through discussions, debates, feedback, and peer assessment. Concurrently, these interactions, supported by teacher and AI chatbot scaffolding, may support EL development.

### Situated cognition theory

Situated Cognition Theory, utilized in this study to construct learning strategies, posits that learning occurs within social, cultural, and physical contexts [31]. It highlights authentic learning in real-world settings, where information is integrated into learners’ experiences through real-life interactions [32,33]. Key principles include authentic resources, collaborative learning, reflection, and scaffolding, as highlighted by Herrington and Oliver (2000) [34].

**Fig 1:**
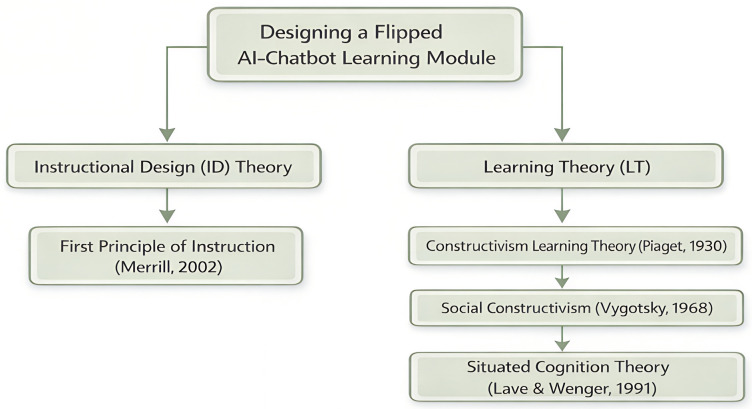
Theoretical Background of the FACL Module.

Notably, this theory is incorporated into the FACL module to support EL development by linking environmental concepts to students’ real-world situations and applying this understanding to decision-making. Simultaneously, AI-powered chatbots can simulate environmental pollution scenarios, making environmental education more engaging by connecting it to students’ local contexts. As such, this increases students’ focus and involvement. Fig 1 illustrates the full FACL module theory.

## Research method

This study focuses on offline curricula in environmental education within the context of higher education in China. The study utilized the Fuzzy Delphi Method (FDM) to identify the primary components of the FACL module. Specifically, FDM is more efficient than the traditional Delphi method, as it saves time and money by using a single questionnaire and eliminating the need for multiple interviews. For instance, Saido et al. (2018) observed that reducing the number of rounds significantly decreased subjectivity and inaccuracy. In addition, FDM converts inherently ambiguous expert judgements into quasi-objective quantitative indicators [35]. Similarly, Wu et al. (2014) noted that this method helps to resolve controversial judgments. In essence, FDM was selected for designing the FACL module due to its effectiveness in aiding decision-making and its reliance on expert input [36].

### Research procedure

The data collection process consisted of two rounds to gather expert opinions. Initially, semi-structured interview questions were developed after a thorough review of literature on EL, AI chatbots, FL, curriculum design theory, relevant government publications, and existing instructional materials. This interview protocol was then validated by three specialists in Educational Technology and Environmental Sciences. Following this, five experienced experts were consulted to create the FDM questionnaire.

**Fig 2:**
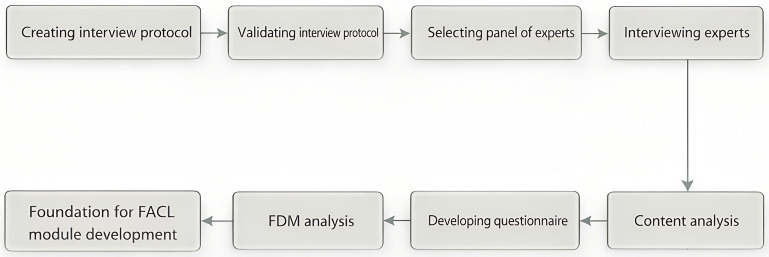
Procedure for FACL module designing.

After finalizing the FDM questionnaire, 12 experts were surveyed to assess their level of agreement. Items with consensus were included in FACL, whereas those without were omitted. This dual-phase method helped ensure that specialists adequately understood the FACL module’s elements. Fig 2 depicts the procedure.

The final findings from the FDM formed the foundation for designing the FACL module. Additionally, the prioritization of selected elements guided the module’s structure.

### Participants

The FDM is primarily concerned with constructing an FACL module intended to support EL development among students by collecting opinions from a group of experts. Note that the selection criteria for the panel’s experts are crucial. This study involved experts with at least five years of experience, selected for their specialized knowledge in education technology, AI, or environmental science. Moreover, practitioners with at least a Master’s degree in a relevant field and familiarity with the Chinese higher education system and its EL requirements were included.

In this context, Adler and Ziglio (1996) suggested that the appropriate number of specialists in a Delphi study may vary depending on the problem’s complexity and the availability of qualified experts. However, they generally recommend a panel size of approximately 10–15 experts, with a minimum of 10 deemed sufficient to achieve stable consensus [37]. Thus, aligning with these guidelines and consistent with previous Fuzzy Delphi studies, in which panel sizes typically range from 10 to 20 experts [38,39], this study recruited 12 experts. Considering the topic’s specialization and expertise requirements, this panel size is both methodologically sound and practically feasible. In response, a snowball sampling technique was employed to identify eligible participants, whereby initial panellists referred additional qualified experts (see Table 1).

**Table 1:** Criteria of experts.

Academic qualifications	Professional experience	Contextual knowledge
Experts have to have at least a Master’s degree in a relevant field (such as education technology, AI, or environmental science).	Experts should have at least 5 years of professional experience in their respective disciplines.	Experts should be familiar with the Chinese higher education system and its EL requirements.

This study was reviewed and approved by the Research Ethics Committee of a university (Protocol No: UM.TNC2/UMREC_3169). All experts were informed of the study objectives and procedures, and their participation was entirely voluntary. Simultaneously, written informed consent was obtained prior to data collection, and all responses were anonymized to ensure confidentiality.

### Instruments

In the initial phase, an interview protocol was utilized with five experts from diverse disciplines, grounded in principles from FPI, Constructivist Learning, Social Constructivism, and Situated Cognition Theory. Subsequently, the insights from these interviews were utilized to develop the FDM questionnaire. Following necessary adjustments, the updated FDM questionnaire was distributed to a group of 12 experts to gain their consensus.

### Data analysis

In this study, data analysis was performed in two stages. First, thematic analysis of the interviews produced the items for the FDM questionnaire. Subsequently, the FDM questionnaire was sent to a panel of 12 experts, and the analysis proceeded through the following steps:

Step 1: Establishing linguistic variables: Each response was assigned three fuzzy values to construct a triangular fuzzy number, reflecting the experts’ degree of fuzziness. These values, m1, m2, and m3, represented the minimum, average, and maximum, respectively, and ranged from 0 to 1. For this purpose, a seven-point scale of linguistic variables was translated into a triangular fuzzy scale (see Table 2).

**Table 2:** Example of a Scale of Linguistic Variables.

	Fuzzy number		
7-point linguistic scale	m1	m2	m3
Strongly Agree	0.90	1.00	1.00
Agree	0.70	0.90	1.00
Somewhat Agree	0.50	0.70	0.90
Neutral	0.30	0.50	0.70
Somewhat Disagree	0.10	0.30	0.50
Disagree	0.00	0.10	0.30
Strongly Disagree	0.00	0.00	0.10

Step 2: Mean Opinions Calculation: This step entails obtaining the mean opinions for each fuzzy value used in the calculation [35,40].

Step 3: Determining the threshold value ‘*d*’: This value is essential for assessing the level of consensus among experts, calculated using the following formula:


$$d(\overset{―}{m},\overset{―}{n})=\sqrt{\frac{\text{1}}{\text{3}}\left[(m\text{1}-n\text{1}{)}^{\text{2}}+(m\text{2}-n\text{2}{)}^{\text{2}}+(m\text{3}-n\text{3}{)}^{\text{2}}\right]}.$$

Step 4: Elements Selection: For all experts, a score of *d* ≤ 0.2 indicates agreement. Additionally, a consensus percentage exceeding 75% is required to confirm a strong agreement among the experts. Items falling below this 75% threshold were excluded. Meanwhile, any item with a fuzzy score (A) below 0.5 was also discarded.

Step 5: The defuzzification process: To confirm the experts’ agreement on the elements of the model’s phases and sub-phases, defuzzification is required, a technique that converts fuzzy numbers into crisp real numbers [41]. The Defuzzification Value (DV) for each questionnaire item is determined using the formula below:


DV=1/3*(m1+m2+m3).

**Fig 3:**
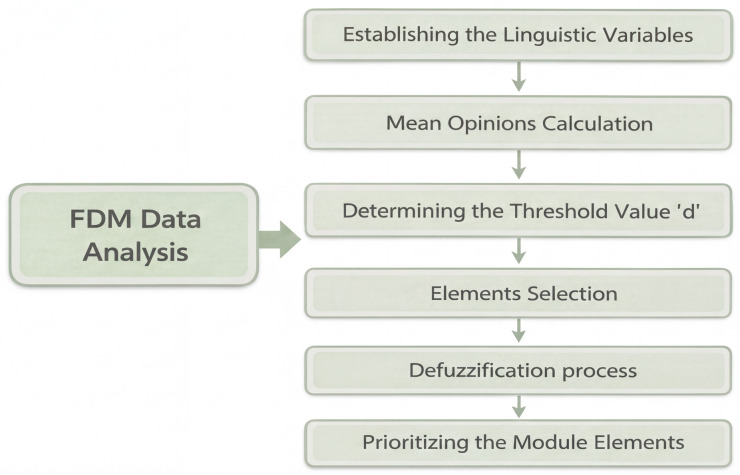
Steps of FDM Data Analysis.

Step 6: Prioritizing the module elements: Elements are ranked according to their DVs for the module’s implementation. The element with the highest DV receives top priority [42] (see Fig 3).

## Results

In the initial phase, the qualifications of the five experts who participated in the semi-structured interviews are detailed in Table 3. These interviews aimed to identify crucial elements for the FACL module. As a result, the analysis yielded seven primary themes: module objectives, content for module design, instructional strategies before class, instructional strategies during class, instructional strategies after class, resources and platforms for delivering the module, and evaluation strategies. These themes informed the creation of an FDM questionnaire, which was later distributed to a group of 12 experts.

**Table 3:** Qualifications of experts for semi-structured interviews (n = 5).

No.	Position	Gender	Educational Level	Experience (Years)	Expertise		
					ES	ET	AI
1	Head of Department In a Public University	Female	PhD	8	**√**		
2	Senior Lecturer in a Public University	Male	PhD	10	**√**		
3	Senior Lecturer in a Public University	Male	PhD	12		**√**	
4	Senior Lecturer in a Public University	Male	PhD	6		**√**	
5	AI industry	Male	PhD	6			**√**

*Notes.* ES = Environmental Science. ET = Educational Technology. AI = Artificial Intelligence.

Table 4 lists the qualifications of the 12 experts involved in the FDM questionnaire. Their agreement on the elements of the FACL module is presented in Tables 5–11.

**Table 4:** Qualifications of Experts Participating in FDM (n = 12).

No.	Position	Gender	Educational Level	Experience (Years)	Expertise		
					ES	ET	AI
1	Head of Department in a Public University	Female	PhD	8	**√**		
2	Senior Lecturer in a Public University	Male	PhD	10	**√**		
3	Senior Lecturer in a Public University	Female	Master	14	**√**		
4	Associate Professor in a Public University	Male	PhD	7	**√**		
5	Associate Professor in a Public University	Female	PhD	8	**√**		
6	Senior Lecturer in a Public University	Male	PhD	12		**√**	
7	Senior Lecturer in a Public University	Male	PhD	6		**√**	
8	Lecturer in a Public University	Female	PhD	5		**√**	
9	Senior Lecturer in a Public University	Male	Master	6		**√**	
10	Senior Lecturer in a Public University	Female	PhD	5		**√**	
11	AI industry	Male	PhD	6			**√**
12	AI industry	Male	PhD	6			**√**

*Notes.* ES = Environmental Science. ET = Educational Technology. AI = Artificial Intelligence.

**Table 5:** Expert Consensus on the Objectives of the FACL Module.

		Triangular Fuzzy Number Process		Defuzzification Process					
No.	Objective	Threshold value(*d*)	Proportion of Expert Consensus, %	m1	m2	m3	Fuzzy Score(A)	Expert Consensus	Ranking
**1**	Promote understanding of fundamental environmental knowledge among students	0.119	91.7	0.750	0.908	0.975	0.878	ACCEPT	**1**
**2**	Enhance the students’ ability to discover and integrate extracurricular resources	0.167	91.7	0.717	0.875	0.958	0.850	ACCEPT	**4**
**3**	Increase students’ awareness and responsibility towards environmental protection	0.169	75.0	0.517	0.717	0.883	0.706	ACCEPT	**8**
**4**	Promote critical thinking and problem-solving skills using emerging AI tools among students	0.174	91.67	0.733	0.883	0.958	0.858	ACCEPT	**3**
**5**	Inspire further action towards environmental conservation among students	0.178	58.33	0.533	0.717	0.875	0.708	REJECT^1^	**7**
**6**	Improve students’ data literacy, helping them make wise decisions based on data analysis	0.187	91.67	0.700	0.858	0.950	0.836	ACCEPT	**5**
**7**	Foster teamwork and communication skills among students	0.171	91.67	0.600	0.783	0.925	0.769	ACCEPT	**6**
**8**	Encourage students to make creative use of generative AI to address environmental challenges	0.143	91.67	0.733	0.892	0.967	0.864	ACCEPT	**2**

*Note*. Essential Criteria: Triangular Fuzzy Numbers: (1) Threshold value (*d*) ≤ 0.2, (2) Expert agreement rate ≥ 75%. Defuzzification Step: Fuzzy score (A) should be ≥ α-cut value of 0.5.

*1*. This objective did not meet the consensus threshold but was later revised and retained in the final module based on theoretical and pedagogical considerations (see Results section).

**Table 6:** Expert Consensus on the Content of the FACL Module.

		Triangular Fuzzy Number Process		Defuzzification Process					
No.	Content	Threshold value(*d*)	Proportion of Expert Consensus, %	m1	m2	m3	Fuzzy Score(A)	Expert Consensus	Ranking
**1**	Introduction of Ecosystems (e.g., fundamentals of ecosystem concepts; ecological balance and destruction of balance; classification of ecosystems)	0.167	91.7	0.717	0.875	0.958	0.850	ACCEPT	**2**
**2**	Soil Ecology (e.g., fundamental knowledge of soil ecology; pollution and self-purification of soil; soil bioremediation)	0.112	100.0	0.750	0.908	0.983	0.881	ACCEPT	**1**
**3**	Air Ecology (e.g., fundamental knowledge of air ecology; hygiene standards for air; detection of air pollution; diseases caused by air pollution; clean air methods)	0.147	100.0	0.700	0.867	0.967	0.844	ACCEPT	**4**
**4**	Aquatic Ecology (e.g., fundamental knowledge of aquatic ecology; polluted water and self-purification; eutrophication; hygiene standards for drinking water)	0.167	91.67	0.717	0.875	0.958	0.850	ACCEPT	**2**

*Note*. Essential Criteria: Triangular Fuzzy Numbers: (1) Threshold value (*d*) ≤ 0.2, (2) Expert agreement rate ≥ 75%. Defuzzification Step: Fuzzy score (A) should be ≥ α-cut value of 0.5.

**Table 7:** Expert Consensus on the Instructional Strategies ‘Before Class’ of the FACL Module.

		Triangular Fuzzy Number Process		Defuzzification Process					
No.	Instructional Strategies-Before Class	Threshold value(*d*)	Proportion of Expert Consensus, %	m1	m2	m3	Fuzzy Score(A)	Expert Consensus	Ranking
**1**	Using the flipped classroom model, in which students pre-learn content through supportive materials (e.g., videos, MOOCs, and online lectures)	0.143	91.7	0.733	0.892	0.967	0.864	ACCEPT	**2**
**2**	Students interact with the AI chatbot for pre-class tasks (reflection questions) and to preview in-class content	0.174	91.7	0.733	0.883	0.958	0.858	ACCEPT	**3**
**3**	Divide students into project groups for tasks. Assign each group a real-world environmental issue to solve, using an AI chatbot for inspiration (e.g., restoring ecological balance or addressing soil pollution)	0.074	100.0	0.817	0.958	1.000	0.925	ACCEPT	**1**
**4**	Students take notes	0.183	66.67	0.533	0.725	0.883	0.714	REJECT	**5**
**5**	After completing pre-class activities, students reflect and come to class with questions	0.139	91.67	0.683	0.867	0.958	0.836	ACCEPT	**4**

*Note*. Essential Criteria: Triangular Fuzzy Numbers: (1) Threshold value (*d*) ≤ 0.2, (2) Expert agreement rate ≥ 75%. Defuzzification Step: Fuzzy score (A) should be ≥ α-cut value of 0.5.

**Table 8:** Expert Consensus on the Instructional Strategies ‘During Class’ of the FACL Module.

		Triangular Fuzzy Number Process		Defuzzification Process					
No.	Instructional Strategies-During Class	Threshold value(*d*)	Proportion of Expert Consensus, %	m1	m2	m3	Fuzzy Score(A)	Expert Consensus	Ranking
**1**	Students participate in whole-class discussions/reviews of pre-class tasks	0.193	66.7	0.583	0.767	0.908	0.753	REJECT	**8**
**2**	The teacher evaluates students’ grasp of pre-class content during class	0.167	91.7	0.717	0.875	0.958	0.850	ACCEPT	**5**
**3**	Lecture given by the instructor with all available multimedia resources (videos, audio, pictures, online resources, PPT slides, interactive quizzes)	0.119	100.0	0.767	0.917	0.983	0.889	ACCEPT	**2**
**4**	Real-world case scenarios are presented during lectures for situated learning	0.122	100.00	0.783	0.925	0.983	0.897	ACCEPT	**1**
**5**	Students discuss new knowledge and ideas with peers	0.160	91.67	0.700	0.867	0.958	0.842	ACCEPT	**6**
**6**	Students’ groups present their problem-solving steps and methods to address real-world issues (group presentation)	0.174	91.67	0.733	0.883	0.958	0.858	ACCEPT	**3**
**7**	The teacher provides guidance and feedback for team/project-based learning	0.136	91.67	0.717	0.883	0.967	0.856	ACCEPT	**4**
**8**	Peers evaluate each other’s groups’ work	0.145	91.67	0.667	0.850	0.958	0.825	ACCEPT	**7**

*Note*. Essential Criteria: Triangular Fuzzy Numbers: (1) Threshold value (*d*) ≤ 0.2, (2) Expert agreement rate ≥ 75%. Defuzzification Step: Fuzzy score (A) should be ≥ α-cut value of 0.5.

**Table 9:** Expert Consensus on the Instructional Strategies ‘After Class’ of the FACL Module.

		Triangular Fuzzy Number Process		Defuzzification Process					
No.	Instructional Strategies-After Class	Threshold value(*d*)	Proportion of Expert Consensus, %	m1	m2	m3	Fuzzy Score(A)	Expert Consensus	Ranking
**1**	Students reflect, linking new knowledge learned before and during class with real-world problems	0.182	83.3	0.650	0.833	0.942	0.808	ACCEPT	**2**
**2**	Students complete assignments/tasks using an AI chatbot or other online resources	0.167	91.7	0.717	0.875	0.958	0.850	ACCEPT	**1**
**3**	After completing assignments, peers evaluate each other’s work	0.182	91.7	0.650	0.825	0.942	0.806	ACCEPT	**3**

*Note*. Essential Criteria: Triangular Fuzzy Numbers: (1) Threshold value (*d*) ≤ 0.2, (2) Expert agreement rate ≥ 75%. Defuzzification Step: Fuzzy score (A) should be ≥ α-cut value of 0.5.

**Table 10:** Expert Consensus on the Instructional Resources and Platforms of the FACL Module.

		Triangular Fuzzy Number Process		Defuzzification Process					
No.	Instructional Resources and platforms	Threshold value(*d*)	Proportion of Expert Consensus, %	m1	m2	m3	Fuzzy Score(A)	Expert Consensus	Ranking
**1**	Lesson plans (teaching plans) for teachers to use	0.126	91.7	0.767	0.917	0.975	0.886	ACCEPT	**2**
**2**	Instructional videos for students to watch before class (Chinese University MOOC, iCourse, ilab-x.com)	0.150	91.7	0.750	0.900	0.967	0.872	ACCEPT	**5**
**3**	Problem-based pre-class task materials for students (reflection questions)	0.126	91.7	0.767	0.917	0.975	0.886	ACCEPT	**3**
**4**	Materials for before-class group tasks (situational questions)	0.112	100.00	0.750	0.908	0.983	0.881	ACCEPT	**4**
**5**	Text-based materials (such as articles, web links, e-books, academic papers, lecture notes)	0.119	100.00	0.767	0.917	0.983	0.889	ACCEPT	**1**
**6**	Post-class tasks/homework	0.189	83.33	0.667	0.842	0.942	0.817	ACCEPT	**7**
**7**	AI chatbot platforms (iFlytek Spark model or ChatGPT)	0.185	83.33	0.733	0.883	0.950	0.856	ACCEPT	**6**

*Note*. Essential Criteria: Triangular Fuzzy Numbers: (1) Threshold value (*d*) ≤ 0.2, (2) Expert agreement rate ≥ 75%. Defuzzification Step: Fuzzy score (A) should be ≥ α-cut value of 0.5.

**Table 11:** Expert Consensus on the Evaluation Strategies of the FACL Module.

		Triangular Fuzzy Number Process		Defuzzification Process					
No.	Evaluation Strategies	Threshold value(*d*)	Proportion of Expert Consensus, %	m1	m2	m3	Fuzzy Score(A)	Expert Consensus	Ranking
**1**	Participation marks as motivation for completing the pre-class task	0.138	100.0	0.683	0.858	0.967	0.836	ACCEPT	**2**
**2**	Assessment of content knowledge: Teacher evaluation (quick Q&A in class)	0.172	91.7	0.633	0.817	0.942	0.797	ACCEPT	**6**
**3**	Assessment of content knowledge: Team presentations	0.173	100.0	0.700	0.858	0.958	0.839	ACCEPT	**1**
**4**	Assessment of critical thinking and problem-solving ability: Team task performance (peer evaluation)	0.153	91.67	0.683	0.858	0.958	0.833	ACCEPT	**3**
**5**	Assessment of content knowledge: Self-reflection	0.196	83.33	0.683	0.850	0.942	0.825	ACCEPT	**4**
**6**	Assessment of critical thinking and problem-solving ability: After-class assignment performance (peer evaluation)	0.196	83.33	0.683	0.850	0.942	0.825	ACCEPT	**4**

*Note*. Essential Criteria: Triangular Fuzzy Numbers: (1) Threshold value (*d*) ≤ 0.2, (2) Expert agreement rate ≥ 75%. Defuzzification Step: Fuzzy score (A) should be ≥ α-cut value of 0.5.

Conversely, Table 12 summarizes the agreed-upon elements for inclusion in the FACL module, intended to inform an instructional framework to support students’ EL development in higher education institutions.

**Table 12:** Summary of the FDM findings for the FACL module based on experts’ consensus.

Elements of the FACL Module	Rank
OBJECTIVE i. Promote understanding of fundamental environmental knowledge among students	1
ii. Encourage students to make creative use of generative AI to address environmental challenges	2
iii. Promote critical thinking and problem-solving skills using emerging AI tools among students	3
iv. Enhance the students’ ability to discover and integrate extracurricular resources	4
v. Improve students’ data literacy, helping them make wise decisions based on data analysis	5
vi. Foster teamwork and communication skills among students	6
vii. Increase students’ awareness and responsibility towards environmental protection	7
viii. Encourage students to participate in real-world environmental actions, such as campus sustainability campaigns or community-based environmental projects	8
CONTENT i. Soil Ecology (e.g., fundamental knowledge of soil ecology; pollution and self-purification of soil; soil bioremediation)	1
ii. Introduction of Ecosystems (e.g., fundamentals of ecosystem concepts; ecological balance and destruction of balance; classification of ecosystems)	2
iii. Aquatic Ecology (e.g., fundamental knowledge of aquatic ecology; polluted water and self-purification; eutrophication; hygiene standards for drinking water)	3
iv. Air Ecology (e.g., fundamental knowledge of air ecology; hygiene standards for air; detection of air pollution; diseases caused by air pollution; clean air methods)	4
INSTRUCTIONAL STRATEGIES-BEFORE CLASS i. Divide students into project groups for tasks. Assign each group a real-world environmental issue to solve, using an AI chatbot for inspiration	1
ii. Using the flipped classroom model, in which students pre-learn content through supportive materials (e.g., videos, MOOCs, and online lectures)	2
iii. Students interact with the AI chatbot for pre-class tasks (reflection questions) and to preview in-class content	3
iv. After completing pre-class activities, students reflect and come to class with questions	4
INSTRUCTIONAL STRATEGIES-DURING CLASS i. Real-world case scenarios are presented during lectures for situated learning	1
ii. Lecture given by instructor with all available multimedia resources (videos, audio, pictures, online resources, PPT slides, interactive quizzes)	2
iii. Students complete group tasks; groups present their problem-solving steps and methods to address real-world issues	3
iv. Teacher provides guidance and feedback for team/project-based learning	4
v. Teacher assesses students’ mastery of pre-class content during class	5
vi. Students discuss new knowledge and ideas with peers	6
vii. Peers evaluate each other’s groups’ work	7
INSTRUCTIONAL STRATEGIES-AFTER CLASS i. Students complete assignments/tasks using an AI chatbot or other online resources	1
ii. Students reflect, linking new knowledge learned before and during class with real-world problems	2
iii. After completing assignments, peers evaluate each other’s work	3
INSTRUCTIONAL RESOURCE AND PLATFORMS i. Text-based materials (such as articles, web links, e-books, academic papers, lecture notes)	1
ii. Lesson plans (teaching plans) for teachers to use	2
iii. Problem-based pre-class task materials for students (reflection questions)	3
iv. Materials for before-class group tasks (situational questions)	4
v. Instructional videos for students to watch before class (Chinese University MOOC, iCourse, ilab-x.com)	5
vi. AI chatbot platforms (iFlytek Spark model or ChatGPT)	6
vii. Post-class tasks/homework	7
EVALUATION STRATEGIES i. Assessment of content knowledge: Team presentations	1
ii. Participation marks as motivation for completing the pre-class task	2
iii. Assessment of critical thinking and problem-solving ability: Team task performance (peer evaluation)	3
iv. Assessment of content knowledge: Self-reflection	4
v. Assessment of critical thinking and problem-solving ability: After-class assignment performance (peer evaluation)	4
vi. Assessment of content knowledge: Teacher evaluation (quick Q&A in class)	6

Table 5 displays the percentage of expert consensus, threshold value ‘*d*’, DV, and rankings for each item. In particular, the table outlines that all experts accepted the following objectives: *promoting understanding of fundamental environmental knowledge among students*; *encouraging students to creatively use generative AI to address environmental challenges*; *promoting critical thinking and problem-solving skills using emerging AI tools*; *enhancing students’ ability to discover and integrate extracurricular resources*; *improving students’ data literacy to make informed decisions based on data analysis*; *fostering teamwork and communication skills* and *increasing students’ awareness and responsibility towards environmental protection*. However, Item 5, ‘*inspire further action towards environmental conservation among students*,’ failed to satisfy all three criteria simultaneously and was excluded. This exclusion reflects the experts’ view that Item 5 was overly general or ambitious compared to the other objectives and lacked specific, actionable steps or measurable outcomes. Nevertheless, the objective’s intention aligns with the widely recognized aims of environmental education. This is particularly true regarding the emphasis on developing learners’ capacity for responsible environmental action. Notably, United Nations Educational, Scientific and Cultural Organization (UNESCO)’s Tbilisi Declaration (1978) explicitly identified ‘participation’ in efforts to improve the environment as one of the five core objectives of environmental education, alongside awareness, knowledge, attitudes, and skills [43]. Although the original wording of this item may have lacked specificity, the underlying purpose remains pedagogically valid. Thus, to preserve this important dimension of environmental education, the objective was retained in the final module, in a revised form that emphasizes concrete, assessable student actions. Accordingly, these actions include participation in campus sustainability initiatives or community-based environmental projects.

Table 6 presents the consensus of content elements for the FACL module. All items received consensus from the experts. They believe the content should include the study of *air, soil, and water ecosystems*, covering land, sea, and air on Earth. These aimed to help learners understand ecosystem functioning and the issues of environmental pollution and protection.

Furthermore, in the instructional strategies ‘before class’ (see Table 7), the majority of experts agreed on several items. These include *dividing students into project groups for discussions and tasks, using the flipped classroom model, where students pre-learn content through supportive materials, having students interact with an AI chatbot for pre-class tasks and to preview in-class content, and, after completing pre-class activities, having students reflect and come to class with questions*. Still, ‘*students take notes*’ received only 66.67% consensus among experts, which was below the 75% threshold and therefore rejected. The likely reason for the rejection is that experts may have considered note-taking a passive activity. In particular, it focuses on passive memory that does not align with the active and proactive learning methods proposed by Constructivist Learning Theory. This emphasizes student engagement and interaction for deeper understanding.

On the other hand, in the instructional strategies ‘during class’ (see Table 8), experts identified *presenting real-world case scenarios during lectures for situated learning* and *lectures given by an instructor with all available multimedia resources (videos, audio, pictures, online resources, PPT slides, interactive quizzes)* as the most essential strategies. Additionally, the following items were accepted by experts: *student groups present their problem-solving steps and methods to address real-world issues (group presentations)*; *teachers provide guidance and feedback for team/project-based learning*; *teachers assess students’ mastery of pre-class content during class*; *students discuss new knowledge and ideas with peers*; and *peers evaluate each other’s group work*. Nonetheless, ‘*students participate in whole-class discussions/reviews of pre-class tasks*,’ which had a 66.7% consensus among experts, was rejected. Experts might have believed that class time is limited and that excessive discussion could lead to ineffective participation and wasted time.

Table 9 presents the consensus of themes for the FACL module. All ‘after-class’ instructional strategies were deemed crucial by the experts. These include *students completing assignments/tasks using AI chatbots or other online resources*, *students reflecting by linking new knowledge with real-world problems,* and *evaluating each other’s work after completing assignments*.

Table 10 presents the experts’ consensus on themes for the FACL module. All items in the ‘instructional resources and platforms’ element were deemed significant for inclusion in the module. In particular, these items include *text-based materials*, *lesson plans for teachers*, *problem-based pre-class task materials for students (reflection questions)*, *materials for before-class group tasks (situational questions)*, *instructional videos for students to watch before class (from platforms like Chinese University MOOC, iCourse, ilab-x.com)*, *AI chatbot platforms*, and *post-class tasks/homework*.

Additionally, Table 11 illustrates the agreed-upon evaluation strategies for the FACL module. The experts agreed on all items in this element. These include *assessing content knowledge through team presentations*, where *participation marks are used to motivate the completion of pre-class tasks*, *critical thinking and problem-solving abilities are evaluated through peer evaluation*, *self-reflection*, and *quick Q&A sessions* in class.

**Fig 4:**
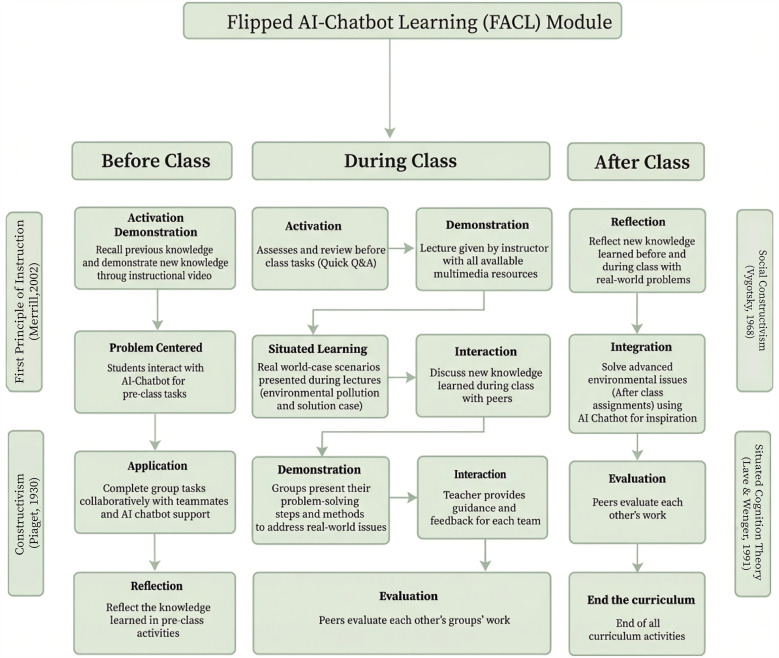
Conceptual structure of the FACL module.

The comprehensive results of the FDM primarily guided the design of the FACL module. Moreover, the hierarchy of the selected elements and sub-elements helped shape the module’s framework. Table 12 and Fig 4 present the conceptual structure of the FACL module derived from the FDM findings.

## Discussion and conclusions

The primary objective of this study was to design an FACL module to support EL development among higher education students by systematically integrating instructional objectives, learning strategies across the before-during-after cycle, instructional resources, platforms, and evaluation strategies. All these are refined through expert consensus using the FDM. Specifically, the instructional objectives included *promoting understanding of fundamental environmental knowledge* and *encouraging students to make creative use of generative AI.* They also aimed to *promote critical thinking and problem-solving skills using AI tools, enhance the ability to discover and integrate extracurricular resources, improve data literacy, and foster teamwork and communication skills.* Additionally, the objectives sought to *increase awareness and responsibility for environmental protection and to encourage participation in real-world environmental actions*. Furthermore, the instructional content consisted of *soil ecology*, *an introduction to ecosystems*, *aquatic ecology*, and *air ecology*. The instructional strategies employed before class included *the flipped classroom model, in which students pre-learned content through supportive materials*, *interacted with an AI chatbot for both individual and group pre-class tasks*, and *reflected on these activities*. During class, strategies included *presenting real-world case scenarios*, *lectures utilizing multimedia resources*, *group presentations, teacher guidance, teacher assessing students’ mastery of pre-class content, discussions*, and *peer review*. After class, strategies focused on *assignments using AI chatbots, self-reflection,* and *peer evaluations*. Instructional resources included *text-based materials*, *lesson plans*, *individual and group pre-class task materials*, *instructional videos*, *AI chatbot platforms, and post-class tasks*. Finally, evaluation strategies encompassed *team presentations*, *participation marks*, *peer evaluations*, *self-reflection*, and *quick Q&A sessions*.

**Fig 5:**
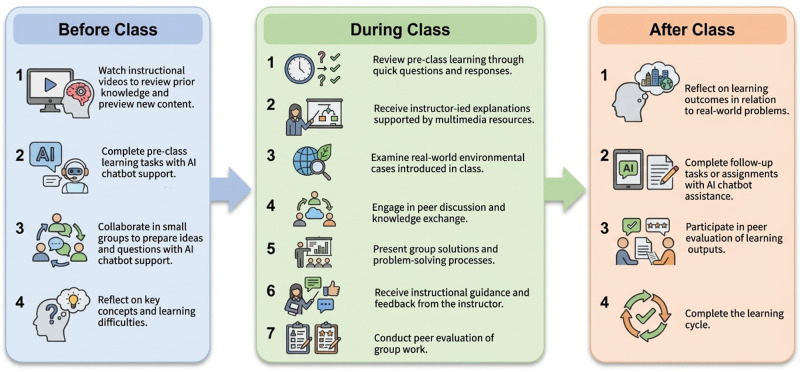
Implementation flow of the FACL module.

To shift from the overall design to its practical enactment, the following section illustrates how the FACL module operates across a typical instructional cycle. Before class, students are assigned a real-world environmental problem (e.g., local soil or water pollution) and complete both individual and team-based preparatory tasks. Individually, students interact with an AI chatbot to answer reflection questions, clarify key concepts, and explore possible solution pathways. Conversely, in teams, students use the chatbot to synthesize ideas, compare perspectives, and develop a shared outline to bring to class. During class, the instructor presents authentic case scenarios and facilitates small-group discussions, providing scaffolding and feedback as students refine their problem-solving approaches. In addition, group presentations and peer discussions are used to consolidate understanding and assess mastery of pre-class content. After class, students complete assignments with AI chatbot support, reflect on real-world applications, and participate in peer evaluation activities. Consequently, through this before-during-after cycle, the FACL module integrates AI-supported preparation with collaborative, instructor-guided learning. A concise summary of this implementation flow is illustrated in Fig 5.

Nonetheless, despite the pedagogical potential of generative AI chatbots, their classroom use also introduces risks that should be acknowledged. Recent studies indicate that large language models may reproduce biases embedded in training data and occasionally generate inaccurate or misleading content. Hence, this raises concerns with regard to equity, accuracy, and trust in educational contexts [44,45]. Moreover, students’ engagement with generative AI varies according to their digital and AI literacy levels, which affects their ability to critically evaluate AI-generated information rather than accept it at face value [46]. If insufficiently guided, the use of AI chatbots may also encourage surface-level engagement or technological dependency, potentially weakening independent reasoning and critical thinking [47]. Specifically, in the FACL module, these risks are addressed by positioning the AI chatbot as a supportive learning aid rather than an authoritative source. Additionally, instructor scaffolding, peer interaction, and structured peer evaluation are embedded to promote critical dialogue, shared reflection, and verification of AI-generated content against credible sources. These strategies aim to foster responsible and reflective use of generative AI in environmental education. Still, addressing these challenges, including both responsible AI use and practical access constraints, requires technological safeguards and deliberate pedagogical guidance.

Furthermore, accessibility and digital equity should be considered when implementing the FACL module across diverse higher education contexts. Students’ access to suitable devices, stable internet connectivity, and institutional digital infrastructure may vary, which can influence participation and learning outcomes. Such disparities have been widely discussed in the digital divide literature. This demonstrates that uneven access and connectivity can amplify educational inequalities in technology-enhanced learning settings [48]. Accordingly, the FACL module can be implemented with a low-threshold logic. Core learning activities rely primarily on text-based chatbot interaction that can be completed on commonly available devices (e.g., smartphones) or shared facilities. Moreover, instructors can provide low-bandwidth or offline alternatives (e.g., downloadable materials, asynchronous completion windows, or group-based access arrangements) for students with limited connectivity. Notably, the module’s collaborative design can further reduce individual access barriers by enabling resource sharing and peer support within teams. This helps students with lower digital access or AI experience remain engaged. Overall, these practical equity-oriented adjustments complement the pedagogical safeguards discussed above. It is intended to help ensure that the FACL module remains inclusive and applicable across institutions with different levels of digital readiness.

Conversely, while AI chatbots provide valuable technological affordances, the effective functioning of the FACL module relies heavily on the instructor’s pedagogical role. In this framework, instructors act as learning designers and facilitators rather than content transmitters. Prior to class, instructors design problem-driven tasks, set expectations for appropriate AI use, and guide students in formulating meaningful questions for chatbot interaction. During class, instructors scaffold learning by prompting critical dialogue, moderating group discussions, and helping students compare AI-generated suggestions with domain knowledge and empirical evidence. Concurrently, they manage collaborative learning dynamics by clarifying group roles, supporting equitable participation, and addressing misconceptions that emerge from peer or AI-supported discussions. After class, instructors provide formative feedback, guide reflective activities, and support peer evaluation processes to consolidate learning and promote deeper understanding. Hence, through these pedagogical practices, the instructor ensures that technological tools are integrated purposefully. This approach maintains instructional coherence while fostering critical thinking, collaboration, and responsible use of generative AI. In essence, these pedagogical roles are grounded in well-established LTs that inform the design and implementation of the FACL module.

Consistent with the theoretical foundations outlined earlier, the FACL module integrates problem-centered design, active knowledge construction, social interaction, and authentic learning contexts to support EL development. Specifically, Merrill’s FPI, which focuses on problem-centered learning and the application of knowledge to real-world issues, provided a strong framework for designing the instructional environment. On the other hand, Constructivist Learning Theory emphasizes active learning and knowledge construction, which aligns well with the flipped classroom approach. Moreover, Social Constructivism highlighted the significance of social interactions and cultural contexts in learning, supporting the collaborative and interactive nature of the FACL module. Additionally, AI and teachers acting as scaffolding may support students’ EL development. Situated Cognition Theory underscores the importance of real-world contexts, aligning with the goal of solving real-world environmental problems through EL. Therefore, building on these theoretical foundations, the study’s findings can be further contextualized by comparing them with existing research in environmental education and educational technology.

The research findings for this study were closely aligned with those of prior studies in the field. For example, the highest consensus for objective elements was to ‘promote understanding of fundamental environmental knowledge among students.’ These findings align with the objectives of environmental education proposed by the Chinese Ministry of Education in 2017. It aims to equip students with the knowledge and skills for harmonious coexistence with the environment [49]. Following this, Esteban Ibáñez et al. (2020) asserted that environmental education should equip students with knowledge to understand and address the Sustainable Development Goals (SDGs) [50].

In particular, for the content elements, experts reached agreement on four themes: ecosystems, soil ecology, aquatic ecology, and air ecology. The prioritization of ecosystems, soil, water, and air within the FACL module is informed by expert consensus. This prioritization is also closely aligned with established theoretical, curricular, and policy frameworks in environmental education and sustainable development. At the systems level, ecosystem-based perspectives emphasize the interdependence of biotic and abiotic components, providing an integrative foundation for understanding environmental change and human-environment interactions [51]. On the other hand, from a policy standpoint, these four ecological domains directly correspond to multiple targets within the United Nations SDGs, particularly SDG 6 (Clean Water and Sanitation), SDG 13 (Climate Action), SDG 14 (Life Below Water), and SDG 15 (Life on Land) [52,53]. Soil health underpins food security and terrestrial ecosystem functioning, aligning with SDG 2 (Zero Hunger) and SDG 15 (Life on Land) [54]. Concurrently, air quality is closely linked to climate regulation and human well-being, corresponding to SDG 3 (Good Health and Well-being) and SDG 13 (Climate Action) [55]. By organizing instructional content around these core ecological systems, the FACL module reflects internationally recognized priorities and ensures curricular relevance to global sustainability agendas. Remarkably, this alignment strengthens the educational validity of the selected content and supports the development of transferable EL competencies. At a more specific level, this policy-aligned rationale is also reflected in experts’ prioritization of particular ecological domains. Notably, the highest consensus was on ‘soil ecology.’ This aligns with Wali et al. (2009), who underscored the significance of ecosystem structure and function, including soil, aquatic, and air ecology, in addressing environmental issues and sustainability [56]. Additionally, Al-Juthery et al. (2023) highlighted the importance of soil health and land quality in environmental restoration and the development of sustainable ecosystems [57].

Specifically, the highest expert agreement on the instructional strategies for the ‘pre-class’ element was ‘grouping students for project work, assigning real-world environmental challenges, and leveraging AI chatbots for inspiration.’ This strategy fosters interaction among students and between students and AI, facilitating the solution of real-life environmental problems. It also aligns with Shih and Tsai’s (2017) findings that group presentations foster teamwork and autonomous learning [58]. Additionally, Li et al. (2022) observed that using chatbots for collaborative activities was associated with improved the learning experience and promotes student engagement [59]. For instructional strategies for the ‘during-class’ element, experts considered the most important element to be ‘presenting real-world case scenarios.’ Shen and Chang’s (2023) study supported this strategy, which revealed that real-world classroom experiences enhance learning motivation and career preparation [60]. Moreover, in terms of instructional strategies for the ‘after-class’ element, experts reached their highest consensus on ‘AI chatbot tools for assignments.’ This finding is proved by Lee et al. (2022), who discovered that using AI chatbots after class was associated with improved academic performance, self-efficacy, and motivation [61].

Generally, all experts agreed that ‘diverse text-based materials, including textbooks, articles, web links, e-books, academic papers, and lecture notes’ were the most critical element in instructional resources and platforms. This agreement aligns with Ramadhani and Fitri’s (2020) findings, which suggested that combining text with multimedia yields better learning outcomes [62]. Similarly, Lo (2017) emphasized that pre-class text-based materials enable more interactive class activities [63].

Regarding the evaluation strategy element, experts recommended ‘assessing students’ content knowledge through team presentations.’ Adnan et al. (2019) supported this approach, noting that formative assessments such as presentations enhance higher-level thinking and soft skills. Notably, collaborative work builds teamwork, communication, and self-confidence, aligning with the social nature of millennial students and preparing them for workplace challenges [64].

Overall, this research’s findings make substantial contributions to the field of environmental education. Theoretically, this study enriches the literature on EL, AI chatbots, and FL by validating the integration of multiple educational theories, FPI, Constructivist Learning Theory, Social Constructivism, and Situated Cognition Theory, into curriculum design. Practically, it develops a comprehensive FACL module that is intended for implementation in higher education institutions, providing educators with a detailed framework intended to support EL development through innovative instructional strategies and AI technologies. Furthermore, the study supports the shift from teacher-centered to student-centered learning. It promotes active and interactive learning environments that align with China’s educational reform initiatives and contribute to the modernization of educational practices.

Beyond theoretical and practical contributions, this study advances multiple domains, including environmental science, educational technology, environmental and information literacy, and the intersecting fields of information and learning science. Particularly, in environmental science, the study addresses the urgent need to support EL development among higher education students. The proposed FACL module encourages students to actively engage with real-world environmental issues, promoting critical thinking and problem-solving skills essential to sustainability. Thus, by bridging theoretical knowledge with practical application, the study supports global sustainability efforts. Additionally, it equips students with the skills required to address complex environmental challenges.

Moreover, in the field of educational technology, the research introduces an innovative integration of generative AI chatbots into the FL model, demonstrating how advanced AI can enhance interactivity, personalization, and adaptability. This integration addresses key challenges in technology-enhanced learning, such as low engagement during pre-class activities, and provides a model for future technological advancements in education. Simultaneously, the framework highlights how generative AI can enhance interactivity, personalization, and formative feedback in the teaching process.

Moreover, the study contributes to both EL and information literacy by aligning instructional strategies with these critical competencies. The module enables students to acquire environmental knowledge while developing essential information literacy skills, including evaluating sources, interpreting data, and making informed decisions. Thus, this dual focus ensures students are equipped to critically and responsibly engage with environmental content, preparing them to navigate a rapidly evolving information landscape.

The study further contributes to information and learning sciences by offering a cohesive framework that links knowledge acquisition with meaningful learning practices. At the same time, the module emphasizes active, student-centered engagement, reflecting the principles of learning science. Specifically, its reliance on generative AI aligns with the core tenets of information science, which prioritize the effective use of technology to facilitate learning. This interdisciplinary convergence enriches theoretical discourse and provides actionable insights for implementing innovative educational solutions.

Lastly, the module is intended to strengthen students’ understanding of and engagement with environmental issues, which may contribute to the development of environmentally literate learners.

In contrast to prior studies that typically examine FL or AI-chatbot-based approaches in isolation [65,66], the FACL module offers a distinct contribution. In particular, it integrates generative AI as a structurally embedded learning scaffold across all instructional phases rather than as a peripheral support tool. However, traditional FL often relies on static pre-class materials and assumes high learner autonomy, whereas many AI chatbot applications are implemented as isolated tutoring or question-answering systems. By contrast, the FACL module aligns AI-supported pre-class preparation, in-class collaborative inquiry, and post-class reflection within a coherent ID grounded in established LTs. This integration enables AI chatbots to support information access and problem framing, peer collaboration, and reflective learning. As such, it strengthens the pedagogical coherence of flipped instruction.

Building on this integrated ID, the FACL module also suggests potential adaptability beyond the Chinese higher education context. Its core design logic is not bound to specific curricula, platforms, or national policies. Instead, it emphasizes a flexible before-during-after learning cycle, pedagogical scaffolding through AI chatbots, and problem-centered, collaborative learning. This flexibility allows the module to be contextually adapted to environmental topics, the language of instruction, and selected AI tools. This makes it applicable to institutions with diverse technological infrastructures and educational traditions. In this regard, the FACL framework may serve as a transferable design reference for educators seeking to integrate generative AI into FL across different cultural and institutional settings.

The FACL module stands at the intersection of generative AI, FL, and EL, offering an innovative framework that addresses critical gaps in traditional education. Unlike other models, it positions AI chatbots as central facilitators of adaptive, real-time guidance and feedback, rather than merely tools for engagement. Simultaneously, this personalized support effectively prepares students before class while sustaining their active participation during and after class. This, in turn, bridges the gap between theoretical knowledge and practical application. Furthermore, its distinctiveness lies in the seamless integration of generative AI within a tri-phased ID that holistically achieves learning needs. Through pre-class preparation, in-class collaboration, and post-class reflection, the module creates a logical progression from knowledge acquisition to critical thinking and actionable environmental behaviors. Interestingly, each phase is meticulously aligned with specific learning objectives, transforming traditional, passive education into a dynamic, outcome-driven process.

Nonetheless, despite its contributions, the present study has several limitations that warrant clearer articulation. (i) The instructional framework was refined through expert consensus using the FDM. This approach is well aligned with the study’s design-oriented purpose and provides a systematic basis for identifying and prioritizing module elements. Despite this, comparable to other consensus-based methods, it primarily reflects experts’ collective judgments about importance and feasibility within the FDM procedure. (ii) The FACL module was developed within the Chinese higher education context, and its direct adoption in other settings may require contextual adaptation. (iii) The study focused on design development and expert validation, and did not include classroom-based evaluation of student learning outcomes. Therefore, learning effectiveness was intentionally positioned as a subsequent stage of investigation.

*Reliance on expert consensus.* The use of FDM enabled the study to consolidate experienced educators’ and experts’ perspectives into a coherent, theory-aligned module structure and a prioritized set of instructional components. Still, although expert consensus is valuable for design validation, it does not fully capture the complexity of implementation conditions across diverse classrooms and institutions. Even when experts are themselves teacher experts with rich practical knowledge, real-world enactment may involve additional constraints and variations. These include factors such as time allocation, class size, learner heterogeneity, institutional arrangements, and access to technology, which cannot be fully represented in a structured consensus procedure. Accordingly, the present findings are best understood as design- and theory-confirming. It provides a well-justified instructional blueprint and a prioritized component set, while acknowledging that implementation experiences may further refine component specifications. Hence, to address this limitation, future research can strengthen the framework through expanded stakeholder validation and classroom-grounded refinement. For instance, subsequent work may include a broader range of participants (e.g., additional teacher experts from different institutions, instructional designers, and technical support staff) to further enrich perspectives on feasibility. It may also incorporate structured feedback from intended users (teachers and students) through interviews, focus groups, and usability-oriented trials of key learning tasks and chatbot interactions. Finally, pilot teaching cycles could be conducted to iteratively optimize activity sequencing, workload, and support strategies under typical instructional constraints. Essentially, these steps would help align expert-endorsed design decisions with classroom realities while preserving the framework’s theoretical coherence.

*Specific context of Chinese higher education*. The module was developed in close connection with Chinese tertiary education conditions, including local curricular emphases, classroom norms, institutional infrastructures, and relevant policy and governance considerations. Thus, such contextual grounding is a strength for relevance and applicability within the target setting. However, it may limit immediate transferability in an unmodified form to other regions where course structures, assessment cultures, language environments, platform availability, or institutional policies differ. Therefore, the framework should be viewed as transportable in principle but adaptable in practice, with attention to local conditions. In response, to enhance cross-context applicability, future studies may adopt a structured adaptation pathway. Specifically, key strategies include pedagogical alignment, retaining the before-during-after cycle while adjusting task difficulty, scaffolding intensity, pacing, and assessment formats to match local course goals and class sizes. At the same time, technological alignment involves selecting chatbot tools compatible with institutional data policies and language needs, and preparing practical alternatives (e.g., LMS-based discussions or prompt templates) when access is constrained. On a similar note, contextual alignment focuses on co-developing cases and projects that reflect local environmental issues and community priorities. Such a staged adaptation, followed by classroom-based evaluation in the new setting, can clarify how the framework performs across educational systems and strengthen its broader relevance.

*Absence of direct measurement of student learning outcomes*. This study concentrated on the development and expert validation of the FACL module components and structure. Consistent with this scope, student outcome evaluation was not included in the current investigation. As a result, the study contributes a theoretically grounded and expert-validated instructional framework, while recognizing that future classroom studies are necessary to examine learning-related impacts and practical implementation experiences. In subsequent research, outcome evaluation can be conducted using validated EL measures that assess knowledge, attitudes, and behavioral orientations. Complementary evidence from performance tasks, such as the quality of problem-solving artifacts, reflective writing, and evidence-based reasoning in projects. Additionally, mixed-method designs can further enrich interpretation by combining quantitative measures with qualitative data. Such data may include teacher reflections, student perceptions, and classroom observations to understand how and why the module supports learning in different instructional contexts. Over multiple cycles of classroom use, these studies can also inform iterative refinements to activity design and chatbot-supported scaffolding. This ultimately strengthens both effectiveness-related understanding and practical usability.

In conclusion, this study introduces a novel FACL framework designed to support EL development among higher education student. By utilizing AI chatbots and the FL model, the module offers a student-centered, interactive, and engaging learning experience. Built on strong theoretical foundations and aligned with current educational trends and policies, the FACL module demonstrates significant potential to support EL development and promote sustainable development. As the current findings stem from expert consensus rather than empirical testing, the conclusions should be regarded as theory-confirming rather than effect-confirming. Accordingly, further quasi-experimental or classroom-based research is required to examine the module’s practical effectiveness on learning outcomes such as environmental knowledge, attitudes, and behavior. Hence, future research and practical application can further validate and refine this innovative approach, aiding the continuous advancement of environmental education.

## Future implementation and piloting strategy

To increase the applied value of the FACL module, future work will involve an extended implementation and piloting plan in actual higher education settings. With assistance from environmental science and educational technology colleagues at partner sites, the module will be integrated within undergraduate modules as part of a semester-long course. Considering this, the delivery will adopt a combined mode consisting of face-to-face activities, the flipped classroom method, and AI-powered chatbot-based pre-class learning activities and post-class formative testing.

In particular, the pilot implementation will occur in three phases: (1) preparatory alignment of the course objectives to the FACL framework, (2) deployment of the module in the classroom over six to eight weeks, and (3) a comprehensive evaluation phase. As such, the evaluation will employ mixed methods. Quantitatively, changes in students’ environmental knowledge, data literacy, and problem-solving skills will be measured before and after the intervention using validated instruments. Following this, the primary effectiveness outcome will be a composite EL index comprising knowledge, attitudes, and behavior, measured using a standardized scale. On the other hand, secondary outcomes will include AI-supported critical thinking and data literacy. Meanwhile, analyses will report gains from baseline and, where a comparison group is available, employ difference-in-differences estimations with effect sizes to assess the practical significance. Furthermore, qualitatively, student learning journals, chatbot interaction logs, and focus group interviews with lecturers will be analyzed to understand student engagement, lesson usability, and the feasibility of implementation.

Overall, particular attention will be paid to institutional suitability, trainers’ readiness, and students’ AI skills. Notably, lessons from this pilot will inform necessary adjustments to content granularity, the depth of chatbot scaffolding, and integration strategies across different teaching formats. Building on this, iterative refinement based on empirical findings will help transform the FACL module into a robust, scalable, and adaptable teaching innovation. Essentially, this process contributes to both the integration of generative AI and EL development in higher education.

## Supporting information

S1 Data Data. (XLSX)
